# Index to the Transactions of the Epidemiological Society of London

**Published:** 1857-04

**Authors:** 


### INDEX.

---

Babington, Dr. B. G., introductory address, 73

Cholera in the island of Fogo, 41

Climate of the Crimea, Dr. Smart on, 1

Epidemic diseases, Dr. Greenhow on study of, 77

Epidemiological Society, quarterly reports of proceedings, 24, 39, 72

Eruptive fevers, Dr. Tripe on mortality from, 25

Gaol fever, Dr. Webb's historical account of, 63

Greenhow, Dr. E. H., study of epidemic diseases, 77

Introductory address by Dr. Babington, 73

Leaõ, J. F. da Silva, cholera in the island of Fogo, 41

Smart, Dr. W. E., climate of the Crimea, 1

Tripe, Dr. J. W., mortality from eruptive fevers, 25

Webb, Dr. F. C., historical account of gaol fever, 63

